# Novel Combretastatin A-4 Analogs—Design, Synthesis, and Antiproliferative and Anti-Tubulin Activity

**DOI:** 10.3390/molecules29102200

**Published:** 2024-05-08

**Authors:** Marta Jędrzejczyk, Benedetta Morabito, Barbara Żyżyńska-Granica, Marta Struga, Jan Janczak, Maral Aminpour, Jack A. Tuszynski, Adam Huczyński

**Affiliations:** 1 Department of Medical Chemistry, Faculty of Chemistry, Adam Mickiewicz University, Uniwersytetu Poznańskiego 8, 61-614 Poznań, Poland; marta.jedrzejczyk@amu.edu.pl; 2 Department of Mechanical and Aerospace Engineering, Politecnico di Torino, 10129 Turin, Italyjack.tuszynski@gmail.com (J.A.T.); 3 Chair and Department of Biochemistry, Medical University of Warsaw, Banacha 1, 02-097 Warsaw, Poland; barbara.zyzynska@wum.edu.pl (B.Ż.-G.);; 4 Institute of Low Temperature and Structure Research, Polish Academy of Sciences, Okólna 2, 50-422 Wrocław, Poland; j.janczak@intibs.pl; 5 Department of Biomedical Engineering, University of Alberta, Edmonton, AB T6G 1H9, Canada; 6 Department of Data Science and Engineering, The Silesian University of Technology, 44-100 Gliwice, Poland; 7 Department of Physics, University of Alberta, Edmonton, AB T6G 2E1, Canada

**Keywords:** antiproliferative activity, binding energy, colchicine binding site, combretastatin, tubulin-targeting agent

## Abstract

Combretastatins isolated from the *Combretum caffrum* tree belong to a group of closely related stilbenes. They are colchicine binding site inhibitors which disrupt the polymerization process of microtubules in tubulins, causing mitotic arrest. In vitro and in vivo studies have proven that some combretastatins exhibit antitumor properties, and among them, combretastatin A-4 is the most active mitotic inhibitor. In this study, a series of novel combretastatin A-4 analogs containing carboxylic acid, ester, and amide moieties were synthesized and their cytotoxic activity against six tumor cell lines was determined using sulforhodamine B assay. For the most cytotoxic compounds (**8** and **20**), further studies were performed. These compounds were shown to induce G0/G1 cell cycle arrest in MDA and A549 cells, in a concentration-dependent manner. Moreover, in vitro tubulin polymerization assays showed that both compounds are tubulin polymerization enhancers. Additionally, computational analysis of the binding modes and binding energies of the compounds with respect to the key human tubulin isotypes was performed. We have obtained a satisfactory correlation of the binding energies with the IC_50_ values when weighted averages of the binding energies accounting for the abundance of tubulin isotypes in specific cancer cell lines were computed.

## 1. Introduction

Combretastatins belong to a group of natural compounds obtained from the bark of the *Combretum caffrum* tree. These phenolic compounds are well-known microtubule-targeting agents (MTAs), which induce the inhibition of tubulin polymerization by binding to the colchicine binding sites (CBSs) in cells. Microtubules are tubulin polymer structures, and by virtue of their prominent role in cell division, migration, and intercellular transport, they became important and available targets in chemotherapy [1]. Cancer cells are capable of uncontrolled proliferation, therefore the destabilization of the mitotic process by interrupting tubulin polymerization can lead to programmed cell death, called apoptosis [2]. Combretastatins comprise four series of compounds: series A is bioactive stilbenes, series B is dihydrostilbenes with a non-ethene bridge, series C includes phenanthrenes, and D series consists of macrocyclic lactones. The interest in combretastatins stems from their significant antitumor activity, observed mainly in the compounds of the series A [3].

**Figure 1:**
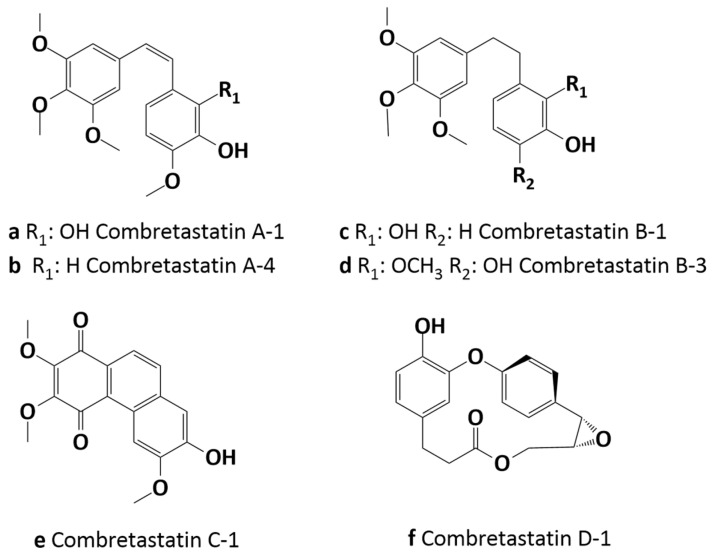
The structures of combretastatins.

Among them, combretastatin A-4 (CA-4, Figure 1, b) deserves the most attention as it was found to show the most potent antimitotic properties evidenced in biological research against the NCI-60 human cancer cell line [4].

**Figure 2:**
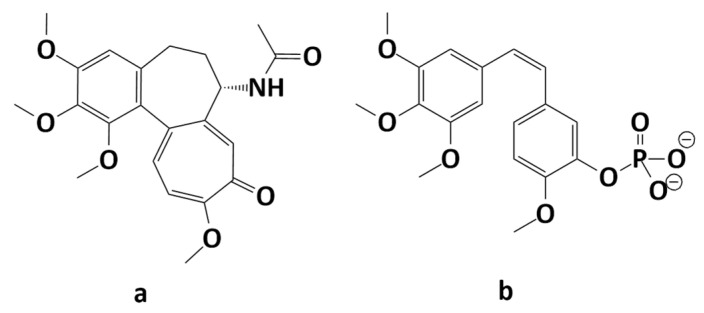
Structures of colchicine (**a**) and combretastatin A-4 disodium phosphate (**b**).

Furthermore, it has been reported that the stereoisomeric configuration of the double bond is related to the biological activity of combretastatins. The combretastatins with cis configuration seem to be significantly more active, while their trans configuration is associated with the deterioration of their biological properties [5]. Combretastatins have structures similar to that of colchicine (Figure 2a), which is also a well-known compound among MTAs. The structures of these two types contain a trimethoxyphenyl ring, while the isovanillinyl group of combretastatins corresponds to the aromatic tropolone ring of colchicine [6]. As a direct consequence, combretastatins exhibit an affinity to the colchicine binding site. Furthermore, the simplicity of their structures permitted the synthesis of many derivatives and biological studies have proved that the chemical modification of combretastatin can lead to compounds with an improved solubility and bioavailability and a higher cytotoxicity [7]. Combretastatin A4 phosphate (C-A4P, Figure 2b) developed by OXiGENE is a good example of an analog which, in contrast to the original compound, is water-soluble [6].

C-A4P is a prodrug which has been tested in preclinical and clinical trials and has been proven to exhibit antitumor properties against anaplastic thyroid cancer in the treatment with carboplatin/paclitaxel combination [8]. It has also been reported that the chemical modification of the ethene bridge can lead to potentially active analogs; therefore, in this study, we focus on the rational modification of the ethene bridge [9,10].

In our study, we synthesized twenty new derivatives of CA-4: one carboxylic acid, six esters, and thirteen amides. The antiproliferative activity of each obtained compound was tested in comparison to that of unmodified combretastatin A-4, colchicine, and podophyllotoxin, which also belong to the group of microtubule-targeting agents. Biological research was conducted using six cancer human cell lines and a human immortalized keratinocyte cell line. Furthermore, we obtained the crystal form of benzyl ester, whose structure was determined by using the X-ray diffraction (XRD) method. The knowledge of the crystal structure was helpful to identify the configuration of a double bond in our analogs, which is crucial for biological activity. Molecular docking computations were performed to confirm the possibility of the binding of these compounds to the colchicine binding site.

## 2. Results

### 2.1. Chemistry

**Figure 3:**
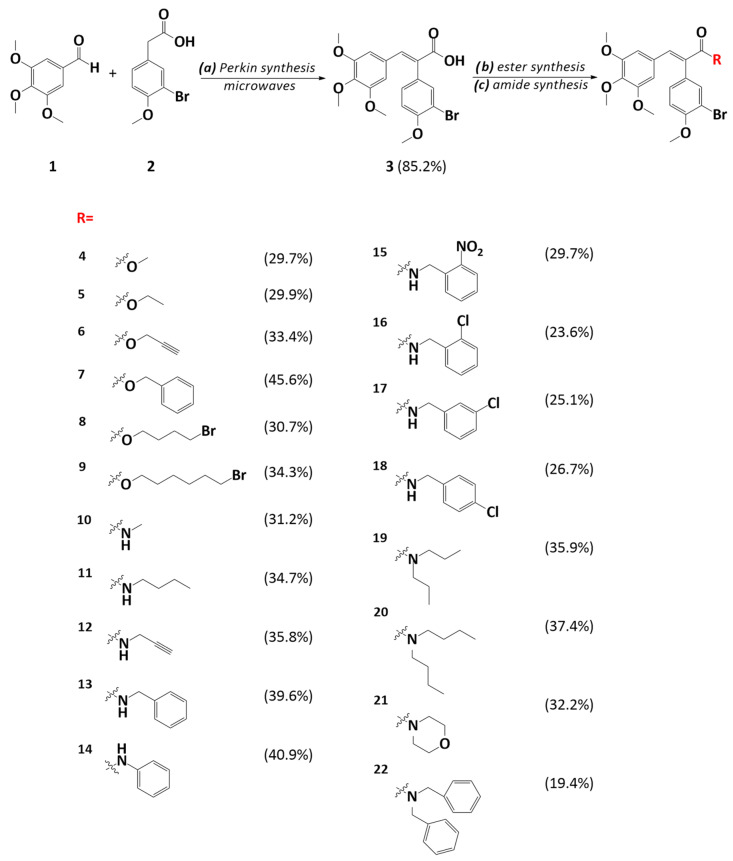
Synthesis of combretastatin A-4 analogs and their structures. Reagents and conditions: (a) triethylamine Et_3_N, acetic anhydride, 120 °C, 30 min, and microwaves; (b) 1,8-diazabicyclo[5.4.0]undec-7-ene (DBU), respective halide, toluene, 100 °C, and 24 h; or *N,N′-*dicyclohexylcarbodiimide (DCC), 4-pyrrolidinopyridine (PPy), *p*-toluenesulfonic acid (*p*-TSA), respective alcohol, CH_2_Cl_2_, 0 °C→r.t., and 24 h; (c) DCC, hydroxybenzotriazole (HOBt), respective amine, CH_2_Cl_2_, and 0 °C→r.t.

It has been proven that the modification of the double bond can improve the biological properties of combretastatin A-4; hence, in our work, we decided to synthesize the esters and amides of CA-4 by modifying the ethylene bridge [10]. To afford those two series of derivatives, at first, we had to conduct Perkin’s condensation using commercially available 3,4,5-trimetoxybenzaldehyde **1** and 3-bromo-4-metoxybenzylacetate acid **2** under microwave irradiation (Figure 3). This reaction led to the formation of the starting compound **3** with a free carboxylic group linked to the double bond. Initially, this reaction was carried out in accordance with the method described by Zou et al. [11]. Their synthetic method consisted of classical heating for 5 h, but after many trials, a condensation product was not observed. We decided to use microwaves to enhance the heating process and reduce the time of reaction. This method turned out to be efficient and crucial for the synthesis of the main substrate **3**.

Two methods of esterification were used for the synthesis of the CA-4 esters. The first method was based on the use of an appropriate alcohol in the presence of N,N′-dicyclohexylcarbodiimide (DCC) as a coupling agent, which activates carboxylic acid and initiates the addition of alcohol. The second strategy of esterification was based on the direct alkylation of the carboxylate ions using respective halides and 1,8-diazabicyclo[5.4.0]undec-7-ene (DBU) as a non-nucleophilic base. This method resulted in higher yields than the previous one. The synthesis of the amides was conducted by using a respective amine in the presence of DCC and hydroxybenzotriazole (HOBt). The structures of the CA-4 esters and amides are shown in Figure 3.

### 2.2. Crystal Structure of Benzyl Ester ***7***

**Figure 4:**
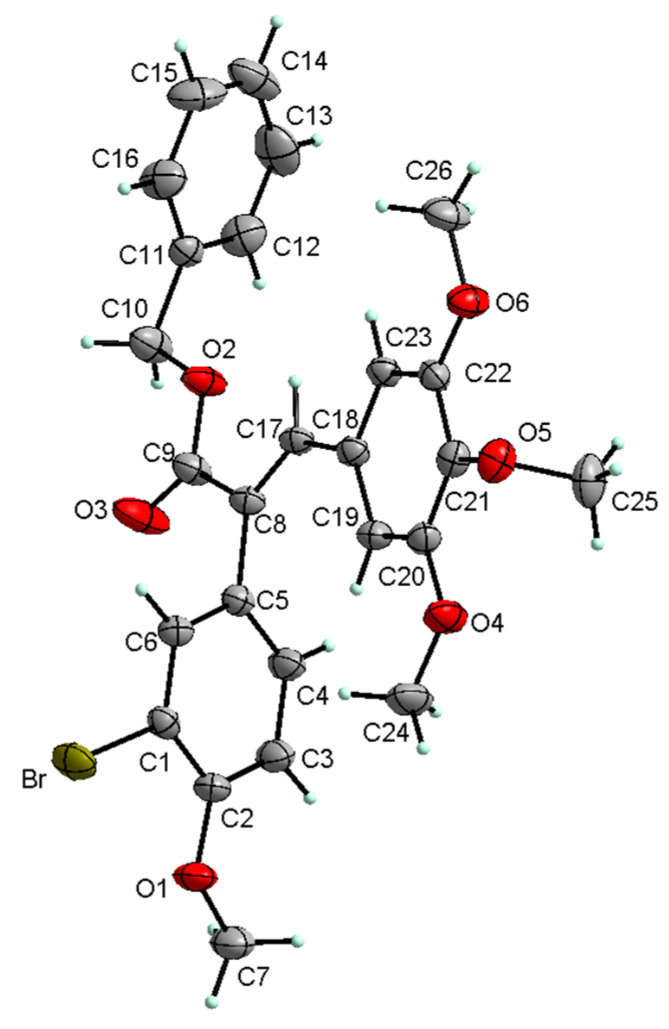
View of the X-ray molecular structure of **7**.

Benzyl ester **7** crystallizes in the non-centrosymmetric space group P21 of the monoclinic system with two molecules per unit cell. The asymmetric unit contains one molecule of **7**. The molecular structure of **7** with the labeling of the atoms is shown in Figure 4. The whole molecule exhibits a non-planar conformation, although it has planar fragments. Two of the three methoxy groups linked to C20 and C22 are almost co-planar with the ring, but the third one at C21 is almost perpendicular to the plane of the ring. In addition, the oxygen atom from the ester group linked via CCH to this ring at C18 is also nearly co-planar with the ring. Both benzene rings, the benzyl ester (C11–C16) and the substituted bromo-methoxy (C1–C6) ones, are almost perpendicular to the fragment of the molecule containing the middle ring with the three methoxy groups. Conformational details can be described in terms of the torsion angles or dihedral angles and are summarized in Table 1.

The arrangement of the benzyl ester **7** molecules in the crystal is mainly determined by the van der Waals forces and the other weak intermolecular interactions. The analyses of the Hirshfeld surface and 2D fingerprint plots are helpful to better understand the nature of the interactions between the components constituting the crystal. The analyses of the Hirshfeld surface (HS) and 2D fingerprint plots are good tools that not only allow a qualitative analysis of the intermolecular interactions in the crystals [12,13,14,15], but also a quantitative analysis, i.e., the determination of the percentage contribution to the HS surface resulting from the particular types of interactions between the molecules in the crystal **7**.

**Figure 5:**
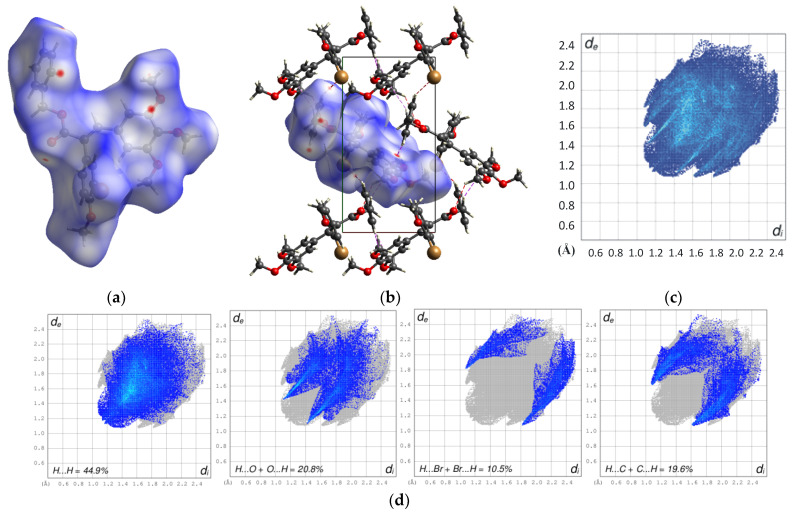
Hirshfeld surface (**a**), the Hirshfeld surface of one molecule with the surrounding ones in the crystal (**b**), the 2D fingerprint plots for the benzyl ester molecule in the crystal **7** (**c**), and the deconvolution of the 2D fingerprint plots of the main interactions (**d**).

The 3D Hirshfeld surfaces are mapped through the normalized contact distance (*d_norm_*) relative to both (*d_e_*) and (*d_i_*) and the van der Waals radii of the atoms, where *d_e_* is the distance from a point on the surface to the nearest nucleus outside the surface, and *d_i_* is the distance from a point on the surface to the nearest nucleus inside the surface and the 2D fingerprint plots of the benzyl ester molecule **7**, and are shown in Figure 5. The sites at which the atoms make intermolecular contacts closer than the sum of their van der Waals radii are marked in red on the HS mapped with the *d_norm_*; the longer contacts are blue and the contacts around the sum of the van der Waals radii are white. The Hirshfeld surfaces clearly show the red spots assigned to the weak C–H‧‧‧O, C–H‧‧‧Br, and C–H‧‧‧Br intermolecular contacts. The contributions of the C–H‧‧‧O, C–H‧‧‧Br, and C–H‧‧‧Br intermolecular interactions to the HS of the benzyl ester molecule are 20.8, 10.5, and 19.6%, respectively. However, the main contribution to the Hirshfeld surface, reaching 44.9%, comes from the H‧‧‧H dispersion forces (Figure 5d). The remaining contributions to the HS (4.2%) comprise those coming from C‧‧‧C (2.0%), C‧‧‧O/O‧‧‧C (1.4%), C‧‧‧Br/Br‧‧C (0.4%), and O‧‧‧Br/Br‧‧‧O (0.4%), which have much less impact on the architecture and arrangement of the benzyl ester molecule **7** in the crystal.

**Figure 6:**
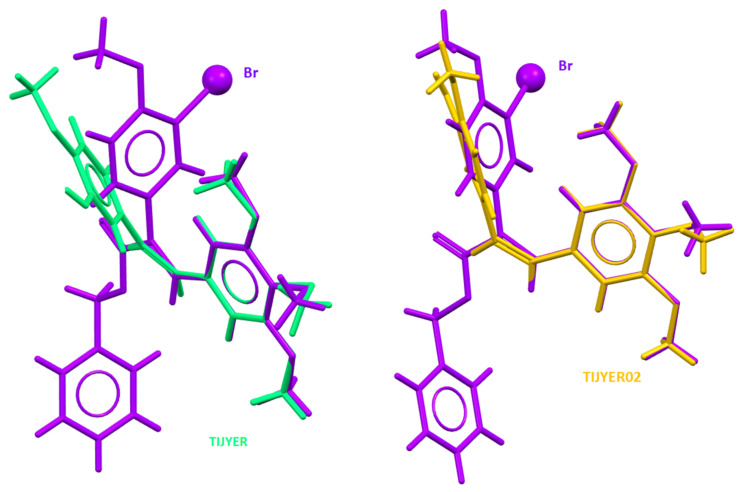
The overlay of the molecular structure of the compound **7** (purple) determined in crystal with the crystal structure of the CA4 from CSD database (corresponding REFCODE in different color in figures).

We compared the molecular structure of the compound **7** to the crystallographic structures of the CA4 previously obtained by other scientists. CA4 is structurally related to colchicine, containing two phenyl rings tilted at 50–60° to each other, linked in cis geometry by a double bond, and containing a 3,4,5-trimethoxyphenyl moiety, which is crucial for targeting the colchicine binding site. The solid-state structure superimpositions of our compound with the two crystal structures of the CA4 shown in Figure 6 demonstrate a significant similarity of common structural motifs. In the solution, the free rotation of the aromatic rings is possible; therefore, these compounds should have a similar orientation at the colchicine binding site.

### 2.3. Biological Results

#### 2.3.1. In Vitro Cytotoxicity

The antiproliferative properties of the studied combretastatin derivatives were tested on six human cancer cell lines, including primary colon (SW480), metastatic colon (SW620), prostate (PC3), liver (HepG2), breast (MDA), and lung (A549) cancer cells, and non-tumor human immortalized keratinocyte cell line (HaCaT) using the sulforhodamine B (SRB) assay. The compounds’ activities were compared to that of the combretastatin A-4 and two other reference compounds: colchicine and podophyllotoxin. As shown in Table 2, most of the synthesized compounds had no effect on cell proliferation (IC_50_ > 100 μM). The most cytotoxic compounds were **8** and **20** characterized by the IC_50_ values between 18.8 and 32.7 µM, depending on the compound and cell line. No selectivity was observed against the tumor cell lines when compared to that against the non-tumor cell line. Nevertheless, these values were much higher than the IC_50_ of the reference compounds (<0.2 μM). Interestingly, the compound **9** with a structure similar to that of the compound **8** and the compound **19** with a structure similar to that of the compound **20** did not show any cytotoxicity.

#### 2.3.2. Cell Cycle Arrest

**Figure 7:**
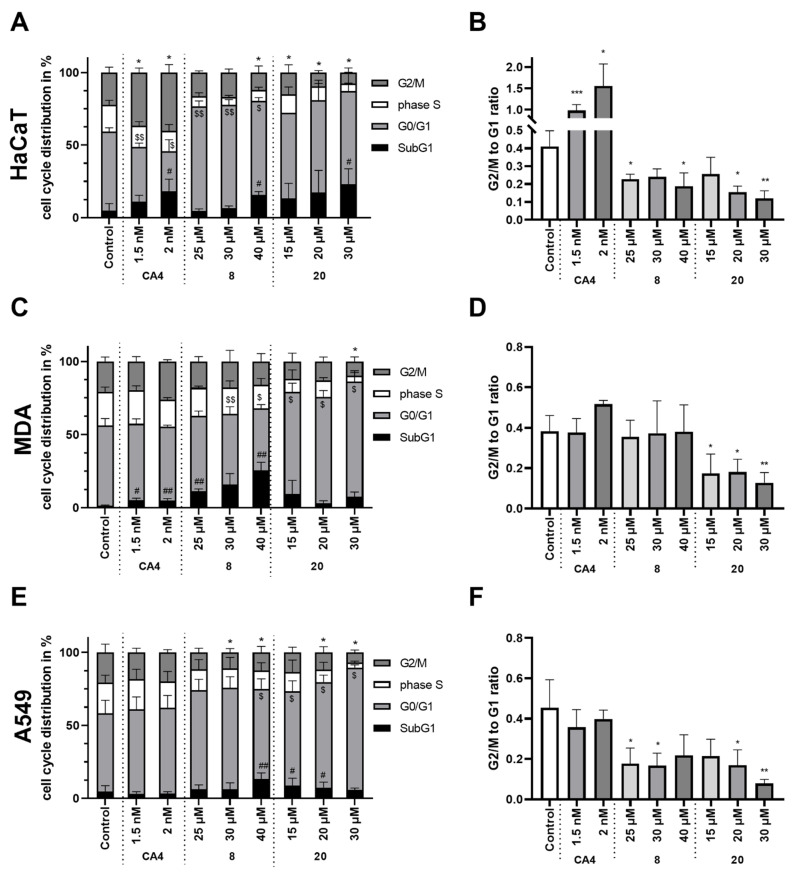
Cell cycle distribution in the HaCaT (**A**,**B**), MDA (**C**,**D**), and A549 (**E**,**F**) cells after treatment with selected compounds for 24 h. (**A**,**C**,**E**) The relative cell cycle distribution of the SubG1, G0/G1, S, and G2/M phases for the HaCaT (**A**), MDA (**C**), and A549 (**E**) cells including the results of the one-way ANOVA analysis with Dunnett’s post hoc of the G2/M (* *p* < 0.05, ** *p* < 0.01, *** *p* < 0.001), G0/G1 ($ *p* < 0.05, $$ *p* < 0.01), and subG1 (# *p* < 0.05, ## *p* < 0.01) proportions between control and treatments; (**B**,**D**,**F**) the ratios of the G2/M and G0/G1 proportion for the HaCaT (**B**), MDA (**D**), and A549 (**F**) cells including the results of the one-way ANOVA analysis with Dunnett’s post hoc in comparison to the control cells (* *p* < 0.05, ** *p* < 0.01).

The impact of the compounds **8** and **20** on the cell cycle phase distribution was assessed and compared to that of the reference compound CA4 (Figure 7). For the compound **8,** G0/G1 phase arrest was observed for the HaCaT (Figure 7B) and A549 (Figure 7F) cells, while for the compound **20,** G0/G1 phase arrest was observed for all three tested cell lines, HaCaT (Figure 7B), MDA (Figure 7D), and A549 (Figure 7F). For the reference compound, CA4, and G2 phase arrest was observed for the HaCaT (Figure 7B) cells, while for MDA and A549 no statistically significant differences were recorded.

Moreover, for the compounds **8** and **20** as well as CA4, the DNA fragmentation represented by the appearance of the subG1 phase was observed for the HaCaT (Figure 7A) cells. For the MDA cells, statistically significant differences between subG1 populations were observed only for the compounds **8** and CA4 (Figure 7C), while for the A549 cells certain differences were noted only for the compounds **8** and **20** (Figure 7E).

For the compound **8** we observed statistically significant differences in the ratio of the percent of cells in the G2/M phase to that of the cells in the G1 phase, for the HaCaT and A549 cells. It was not observed for the MDA cells. However, we can see that the compound **8** had the strongest cytotoxic effect on the MDA cells, observed by comparing the number of cells in the subG1 phase which was growing with the growing concentrations of the compound. The phase subG1 includes cells with a diminished DNA content due to DNA fragmentation and apoptosis. This shows that the compound **8** showed a stronger cytotoxic activity on the MDA cells than the cell cycle arrest effect which was dominating in the HaCaT and A549 cells.

#### 2.3.3. Tubulin Polymerization Acceleration

**Figure 8:**
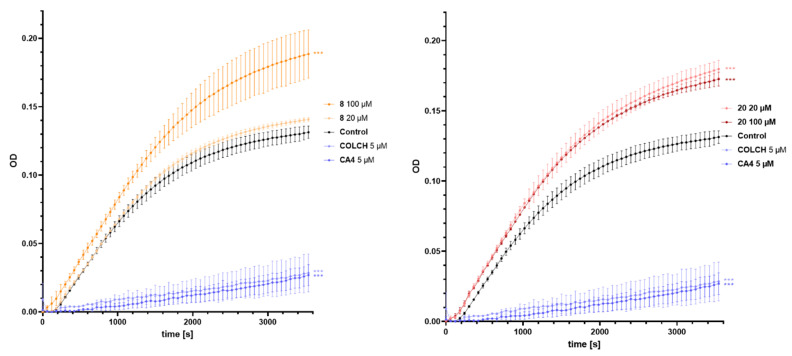
Effect of the selected compounds on the kinetics of in vitro tubulin polymerization. The time dependence of the tubulin polymerization at 37 °C in the presence of vehicle (1% *v/v* DMSO, control, black line) or the selected compounds at different concentrations (colored lines) as indicated, measured by turbidometry at 340 nm. Each turbidometry value represents the mean ± SD from two independent experiments. The results were compared using one-way ANOVA analysis with Dunnett’s post hoc in comparison to the control cells (*** *p* < 0.001).

The effect of the selected compounds on in vitro tubulin polymerization was compared to the activity of the reference compounds, CA4 and colchicine (COLCH). The kinetics of microtubule assembly was assessed by measuring absorbance at 340 nm over time. In contrast to the reference compounds CA4 and COLCH which inhibited tubulin polymerization, both the compounds, **8** and **20**, enhanced tubulin polymerization (Figure 8). For the compound **8** the acceleration was concentration-dependent, while for the compound **20** at both tested concentrations (20 and 100 μM) the acceleration was on a similar level. These results can be compared to the effect of paclitaxel, a well-known representative of tubulin stabilizing agents, on tubulin polymerization. In this study the compounds **8** and **20** exhibited a mode of action very similar to that of microtubule stabilizers [16].

**Table 2:** Cytotoxicity (IC_50_, µM) of studied compounds estimated by SRB assay [17].

Compound	SW480	SW620	PC3	HepG2	MDA	A549	HaCaT
**3**	>100	>100	>100	>100	>100	>100	>100
4 *	57.6 ± 9.4	61.8 ± 11.4	65.9 ± 16.1	63.7 ± 12.6	55.2 ± 17.3	53.0 ± 10.8	68.3 ± 19.6
**5**	73.6 ± 8.4	57.5 ± 3.9	43.2 ± 10.2	42.1 ± 10.2	45.2 ± 5.5	46.6 ± 12.1	45.0 ± 7.2
6 *	>100	>100	>100	>100	>100	>100	>100
**7**	>100	>100	>100	>100	>100	>100	>100
**8**	32.7 ± 6.7	24.2 ± 8.4	31.0 ± 5.5	25.54 ± 6.3	23.1 ± 7.7	19.6 ± 2.0	23.9 ± 3.9
**9**	>100	>100	>100	>100	>100	>100	>100
10 *	52.2 ± 17.9	46.0 ± 11.4	44.9 ± 12.1	50.5 ± 10.5	55.5 ± 20.3	49.3 ± 16.3	55.3 ± 17.0
11 *	50.2 ± 11.2	50.8 ± 14.5	67.2 ± 18.6	50.5 ± 17.5	61.4 ± 21.8	65.1 ± 17.3	58.6 ± 22.2
**12**	>100	>100	>100	>100	>100	>100	>100
**13**	59.8 ± 23.0	62.4 ± 3.1	55.8 ± 23.7	56.2 ± 22.2	59.8 ± 18.8	82.1 ± 13.9	68.5 ± 4.8
**14**	>100	>100	>100	>100	>100	>100	>100
**15**	>100	>100	>100	>100	>100	>100	>100
16 *	>100	>100	>100	>100	99.8 ± 3.9	92.8 ± 7.7	>100
**17**	>100	>100	>100	>100	>100	>100	>100
**18**	>100	>100	>100	89.6 ± 24.4	66.7 ± 6.4	62.2 ± 8.6	>100
**19**	>100	>100	>100	>100	>100	>100	>100
**20**	18.8 ± 0.8	20.9 ± 2.9	23.7 ± 10.3	25.8 ± 9.6	19.0 ± 7.1	19.4 ± 2.2	20.5 ± 9.7
**21**	>100	>100	>100	>100	>100	>100	>100
**22**	>100	>100	>100	>100	>100	>100	>100
**CA4**	0.00281 ± 0.00017	0.00221 ± 0.00088	0.00210 ± 0.00026	0.00158 ± 0.00019	0.00372 ± 0.00027	0.00343 ± 0.00096	0.00427 ± 0.00047
**podophyllotoxin**	0.0444 ± 0.0022	0.0292 ± 0.0078	0.0129 ± 0.0015	0.0258 ± 0.0056	0.0293 ± 0.0028	0.0247 ± 0.0039	0.0316 ± 0.0040
**colchicine**	0.166 ± 0.065	0.130 ± 0.089	0.118 ± 0.047	0.107 ± 0.025	0.110 ± 0.015	0.094 ± 0.009	0.104 ± 0.012

Data are expressed as mean ± SD from at least three independent experiments, IC_50_ (μM)—the concentration of the compound that corresponds to a 50% growth inhibition of the cell line (as compared to the control cells) after 48 h culture with the individual compound * Due to low compound solubility the exact IC_50_ value could not be determined, the real values might be lower.

### 2.4. Computational Studies

To estimate the binding affinity between the new combretastatin A4 derivatives and β tubulin isotypes, the docking procedure was carried out using the Autodock4 software, and the results obtained are presented in Table 3 and Table 4; the binding energy values are expressed in kcal/mol.

The values in Table 3 refer to the binding energies of each compound docked to each tubulin isotype separately. However, different cell lines have distinct expression levels of the tubulin isotypes, which we were able to identify and use for the calculation of the corresponding weighted binding energies accounting for the different distributions of the β tubulin isotypes in the seven tumor cell lines available. The reason for adopting this approach is that within each cell line, various tubulin isotypes coexist albeit at different expression levels and exhibit different affinities to the tested compounds. Therefore, calculating a weighted average of the binding energy that is based on the specific tubulin isotype abundance in a given cell type is a more accurate way to simulate the experimental conditions. This is shown in Table 4 for each of the seven cell lines tested in our experimental assays.

The main purpose of this computational study was to find an explanation, through linear models, of the biological behavior of the new derivatives and obtain insights into their properties. Therefore, a possible correlation was tested between the binding affinity calculated by the docking procedures and the experimental results, particularly, the IC_50_ values obtained independently in the biological assays.

**Figure 9:**
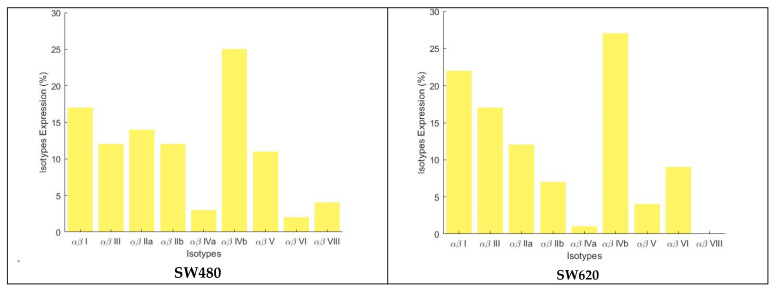

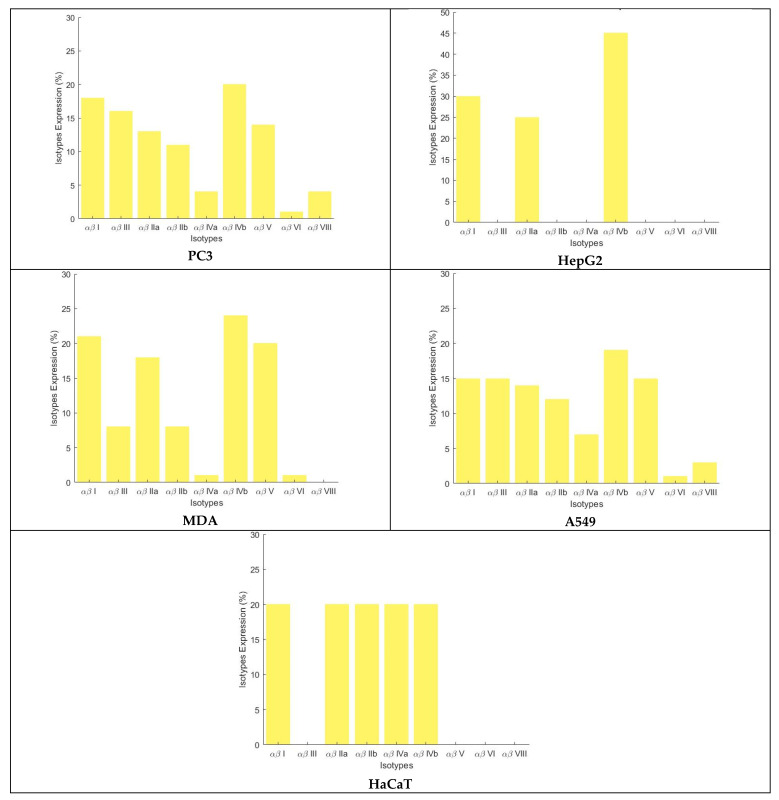
Tubulin isotype relative expression levels in the cell lines investigated in this study.

For the cell lines SW620, MDA, and PC3 it was possible to have the exact gene expression values since the data for these cell lines are present in the CellMiner database. In contrast, for the other cell lines, approximations had to be made to estimate the expression rates of the β tubulin isotypes. For the SW480 and A549 cell lines, the relative percentages of the individual tubulin isotype expression levels were approximated by considering the average gene expression values of all the cancer cells belonging to that particular tissue in the CellMiner database, namely the colon and lung cancer cells, respectively. For the HepG2 cell line, which is a liver cancer cell line, there are no cancer cells of the same organ in the CellMiner database, so it was decided to use the gene expression level of the healthy cells of this tissue, and the data were taken from the work of Garcia et al. [18]. For the HaCaT cell line, i.e., human immortalized keratinocyte cell line, it was not possible to find sufficient data to estimate a precise expression of the isotypes, so in order to obtain the binding energy weighted on the percentage of the isotypes expressed, a strong approximation was made, assuming a uniform distribution over the isotypes usually most expressed in the cells (Figure 9), and these values were also taken from the work of Garcia et al.

Knowing that combretastatin binds to the same binding site as colchicine due to the trimethoxyphenyl part of the molecule, the grid box was centered in the following coordinates for the X, Y, and Z, respectively: −16, 13, and −23. The dimensions of the box along the three dimensions are 40 × 40 × 40, with a spacing of 0.375 Å. The appropriate poses of the compounds docked to the target proteins (tubulin isotypes) were clustered, and the binding energy considered was taken from the most populated cluster.

In our QSAR analysis, we have included the main standard descriptors used in the literature, namely: the topological polar surface area (TPSA), which is largely responsible for the physicochemical properties of the drug, the partition coefficient (MlogP), which characterizes solubility properties, as well as the polarizability of the molecule and the number of hydrogen bond donors (HBDs), in addition to the estimates of the binding free energy, allowing us to quantify the target–drug interactions. The results obtained in our correlation analysis show that the binding affinity appears to be the best descriptor that correlates with the biological behavior of the compounds, while the partition coefficient (MlogP) and the polarizability of the molecule appear to show almost no correlation. These observations are illustrated with Table 5 data.

From the R^2^ values calculated for the obtained models, it is easily seen that the weighted binding energy (BE*_w_*) shows the strongest correlation with logIC_50_, thus with the biological activity of the molecules.

Consequently, the biological activity (cytotoxicity) of the molecules tested is well correlated with the averaged affinity of these compounds to the tubulin isotypes present in the cell lines investigated. As shown in Table 5, we have achieved satisfactory correlation levels for all cell lines, indicating that the tubulin isotype expression weighted average values of the binding energy provide good indicators of the cytotoxic activity of the investigated compounds.

One point to note is that higher correlation coefficient values were obtained with the toxicity data when using the weighted binding energies, calculated by considering the closest to the real expression of the β tubulin isotypes in the cell lines studied than using a hypothetically uniform distribution of the isotypes. Finally, the HaCaT cell line for which the exact percentage distribution could not be found, has the lowest R^2^ value.

## 3. Discussion

For all of the synthesized compounds, their cytotoxicities against the six cancer cell lines and non-cancer keratinocyte cell line were assessed. Surprisingly, most of the CA4 analogs were not cytotoxic in contrast to the high toxicity of the CA4. The highest cytotoxicity among the synthesized compounds was observed for the compounds **8** and **20** and thus these compounds were used for further in vitro analysis. The main mechanism of the CA4 activity is tubulin polymerization inhibition and it results in the cell cycle G2 phase arrest. Thus, the selected compounds’ influence on cell cycle progression was determined by flow cytometry. Unexpectedly, both the compounds did not arrest cells in the G2 phase but caused the G0/G1 cell cycle arrest. Furthermore, when the influence on tubulin polymerization was compared, both the compounds (**8** and **20**) caused the acceleration of this process instead of its inhibition like the CA4. In consequence, their mode of action became very similar to that of paclitaxel. It is not the first time that the CA4 derivatives act as microtubule-stabilizing agents in contrast to the effect of the unmodified compound. In 2013 it was shown for the first time that one of the synthesized CA4 cyclopropylamide analogs can stimulate tubulin polymerization as a stabilizing factor. However, the cell cycle arrest was induced by this compound in a dose-dependent manner. Contrary to the compounds **8** and **20**, the cyclopropylamide analog significantly arrested the cell cycle at the G2/M phase at concentrations of 5 and 10 µM [19].

The computational work performed in this study involved the docking of all the synthesized combretastatin A4 derivatives to the main β tubulin isotypes in order to find their binding sites and binding poses and estimate their binding free energies. While docking calculations seldom provide accurate binding energies, they provide a reliable approximation of the relative binding affinities which can then be compared with experimental data. Importantly, different cell types express β tubulin isotypes differently. For this reason, following our docking simulations, we could account for the tubulin isotype expression levels in different cell lines when performing the linear regression analysis between the computed binding energies and the experimental logIC_50_ data by introducing weighted averages corresponding to the specific isotype expression levels in each cell line. Models were created for the seven cancer cell lines for which the experimental results were provided in this work. The results show that taking into account the correct expression of the isotypes in different cell lines improves the regression models, compared with the model in which a uniform distribution of the most common isotypes is assumed. However, from the docking scores for affinity to specific tubulin isotypes, it also appears that none of the compounds tested exhibits strong selectivity for any of the tubulin isotypes it binds to. Overall, the most promising derivatives indicated by the computational work are **8** and **20**, which bind to tubulin stably over time. Furthermore, from the results obtained by calculating the binding free energy, ∆G_bind_, using the MM/GBSA technique, which is more time-consuming and computationally expensive, but more accurate, it appears that one of the two ligands, namely **20**, exhibits some selectivity to βIII, a tubulin isotype that is commonly overexpressed in cancer cells [20].

## 4. Materials and Methods

### 4.1. Chemistry

#### 4.1.1. General Procedures

All reagents and all solvents were obtained from Merck or Trimen Chemicals S.A. (Poland) and were used as received without further purification. The CDCl_3_ spectral grade solvent was stored over 3 Å molecular sieves for several days. The reaction mixtures were stirred using teflon-coated magnetic stir bars and were monitored by thin layer chromatography (TLC) using aluminum-backed plates 60F_254_ (Merck KGaA, Darmstadt, Germany). The TLC plates were visualized by UV light (254 nm), followed by treatment with phosphomolybdic acid (PMA, 5% in absolute EtOH) and gentle heating. The products of the reactions were purified using the CombiFlash^®^Rf+ Lumen Flash Chromatography System (Teledyne Isco, Lincoln, NE, USA) with integrated ELS and UV detectors. All the solvents used in the flash chromatography were of HPLC grade (Merck) and were used as received. The solvents were removed using a rotary evaporator.

The NMR spectra were recorded on a Varian 400 (^1^H NMR at 403 MHz, ^13^C NMR at 101 MHz) magnetic resonance spectrometer. The ^1^H NMR spectra are reported in chemical shifts downfield from TMS using the respective residual solvent peak as the internal standard (CDCl_3_ δ 7.26 ppm). The ^1^H NMR spectra are reported as follows: chemical shift (δ, ppm), multiplicity (s = singlet, d = doublet, q = quartet, dd = doublet of doublets, dt = doublet of triplets, dq = doublet of quartets, ddd = doublet of doublet of doublets, ddt = doublet of doublet of triplets, dddd = doublet of doublet of doublet of doublets, m = multiplet), coupling constant(s) in Hz, and integration. The significant peaks are reported within the overlapping region ~2.00–0.50 ppm of the ^1^H NMR spectra. The ^13^C NMR spectra are reported in chemical shifts downfield from TMS using the respective residual solvent peak as the internal standard (CDCl_3_ δ 77.36 ppm). The line broadening parameters were 0.5 or 1.0 Hz, while the error of chemical shift value was 0.1 ppm.

The electrospray ionization (ESI) mass spectra were recorded on a Waters/Micromass ZQ mass spectrometer (Waters Alliance) equipped with a Harvard syringe pump. The samples were prepared in dry acetonitrile and were infused into the ESI source using a Harvard pump at a flow rate of 20 mL/min. The ESI source potentials were as follows: capillary 3 kV, lens 0.5 kV, and extractor 4 V. The standard ESI mass spectra were recorded at the cone voltages of 10 and 30 V. The source temperature was 120 °C and the desolvation temperature was 300 °C. Nitrogen was used as the nebulizing and desolvation gas at flow rates of 100 dm^3^/h. The mass spectra were acquired in the positive ion detection mode with unit mass resolution at a step of 1 *m*/*z* unit. The mass range for the ESI experiments was from *m*/*z* = 300 to *m*/*z* = 1100.

#### 4.1.2. Synthesis of Compound **3**

3-bromo-4-methoxyphenylacetic acid (1.0 eq) and 3,4,5-trimethoxybenzaldehyde (1.0 eq) were added to acetic anhydride (7.8 eq) and triethylamine (2.0 eq). The reaction mixture was stirred under 120 °C for 30 min using microwave radiation in the reactor Mars™ 6 (CEM Corporation, Matthews, NC, USA). After cooling, concentrated HCl was added to the mixture to achieve an acidic pH, and the process was controlled using the paper indicator to monitor the pH. Then, the reaction mixture was poured into an ice-water bath, and after 24 h of stirring the precipitate was filtrated under reduced pressure. The residue was dissolved in CH_2_Cl_2_ and then extracted twice with H_2_O to remove the rest of the hydrochloric acid. The organic layers were combined and evaporated under reduced pressure to dryness. The residue was purified by column flash chromatography on silica gel using CombiFlash^®^Rf+ (chloroform/ethyl acetate, increasing concentration gradient) with an integrated Evaporative Light Scattering Detector (ELSD) and UV detector. Yield: 85.2%. ESI-MS for C_19_H_19_BrO_6_ (*m*/*z*): [M + Na]^+^ 445.2. ^1^H NMR (401 MHz, DMSO) δ 7.70 (s, 1H), 7.42 (d, *J* = 1.6 Hz, 1H), 7.20–7.14 (m, 2H), 6.44 (s, 2H), 3.86 (s, 3H), 3.62 (s, 3H), and 3.50 (s, 6H). ^13^C NMR (101 MHz, DMSO) δ 168.23, 154.93, 152.35, 139.63, 138.38, 133.86, 130.61, 130.44, 130.13, 129.50, 112.86, 110.53, 108.07, 60.08, 56.41, and 55.37.

#### 4.1.3. General Procedure for the Synthesis of Compounds **4** and **5**

Compound **3** (1.0 eq) was dissolved in CH_2_Cl_2_ (20 mL) and cooled in an ice-water bath. Then, DCC (1.5 eq) was added, and successively every 15 min the following substances were added: PPy (0.5 eq), *p*-TSA (0.25 eq), and respective alcohol (10.0 eq). The reaction mixture was stirred for 24 h. Then, the precipitated DCU was separated under reduced pressure and the filtrate was evaporated to dryness in the presence of silica gel. The residue was purified by column flash chromatography using CombiFlash^®^Rf+ (chloroform/ethyl acetate, increasing concentration gradient) with an integrated Evaporative Light Scattering Detector (ELSD) and UV detector.

Compound **4**: Yield: 29.7% ESI-MS for C_20_H_21_BrO_6_ (*m*/*z*): [M + Na]^+^ 460. ^1^H NMR (401 MHz, CDCl_3_) δ 7.74 (s, 1H), 7.48 (d, *J* = 2.1 Hz, 1H), 7.26 (s, 1H), 6.34 (s, 2H), 3.90 (s, 3H), 3.81 (s, 3H), 3.79 (s, 3H), and 3.58 (s, 6H). ^13^C NMR (101 MHz, CDCl_3_) δ 167.93, 155.46, 152.63, 140.86, 134.61, 130.11, 129.63, 129.57, 129.40, 112.02, 111.70, 108.08, 60.79, 56.29, 55.64, and 52.37.

Compound **5**: Yield: 29.9% ESI-MS for C_21_H_23_BrO_6_ (*m*/*z*): [M + Na]^+^ 474. ^1^H NMR (401 MHz, CDCl_3_) δ 7.71 (s, 1H), 7.47 (d, *J* = 2.1 Hz, 1H), 7.15 (dd, *J* = 8.4, 2.1 Hz, 1H), 6.94–6.91 (m, 1H), 6.34 (s, 2H), 4.28–4.22 (m, 2H), 3.89 (s, 3H), 3.81 (s, 3H), 3.58 (s, 6H), and 1.29 (t, *J* = 7.1 Hz, 3H). ^13^C NMR (101 MHz, CDCl_3_) δ 167.39, 155.38, 152.60, 140.36, 134.65, 130.15, 129.98, 129.70, 129.49, 111.93, 111.56, 108.02, 61.18, 60.76, 56.26, 55.63, and 14.22.

#### 4.1.4. General Procedure for the Synthesis of Compounds **6**–**9**

Compound **3** (1.0 eq) was dissolved in toluene/DMF (15 mL/5 mL), and then DBU (1.3 eq) was added. Subsequently, the mixture was stirred and heated at 100 °C, and after 30 min the respective halide (3.0 eq) was added. The reaction mixture was stirred and heated at 100 °C for 6 h and then stirred at room temperature for 24 h. The mixture was evaporated to dryness using acetonitrile to remove the toluene and DMF. The residue was purified by column flash chromatography on silica gel using CombiFlash^®^Rf+ (chloroform/ethyl acetate, increasing concentration gradient) with an integrated Evaporative Light Scattering Detector (ELSD) and UV detector.

Compound **6**: Yield: 33.4% ESI-MS for C_22_H_21_BrO_6_ (*m*/*z*): [M + Na]^+^ 485. ^1^H NMR (400 MHz, CDCl_3_) δ 7.71 (s, 1H), 7.42 (d, *J* = 2.1 Hz, 1H), 7.10 (dd, *J* = 8.4, 2.1 Hz, 1H), 6.89–6.86 (m, 1H), 6.29 (s, 2H), 4.73 (d, *J* = 2.5 Hz, 2H), 3.83 (s, 3H), 3.75 (s, 3H), 3.52 (s, 6H), and 2.43 (t, *J* = 2.5 Hz, 1H). ^13^C NMR (101 MHz, CDCl_3_) δ 166.57, 155.50, 152.58, 141.66, 139.21, 134.62, 130.20, 129.18, 129.08, 128.80, 111.96, 111.64, 108.09, 77.69, 74.87, 60.80, 56.25, 55.61, and 52.57.

Compound **7**: Yield: 45.6% ESI-MS for C_26_H_25_BrO_6_ (*m*/*z*): [M + Na]^+^ 536. ^1^H NMR (401 MHz, CDCl_3_) δ 7.76 (s, 1H), 7.51 (d, *J* = 2.1 Hz, 1H), 7.40–7.32 (m, 6H), 7.18 (d, *J* = 8.4, 2.1 Hz, 1H), 6.94 (d, 1H), 6.35 (s, 2H), 5.26 (s, 2H), 3.91 (s, 3H), 3.82 (s, 3H), and 3.59 (s, 6H).^13^C NMR (101 MHz, CDCl_3_) δ 167.23, 155.47, 152.63, 140.94, 136.04, 134.74, 130.22, 129.63, 129.56, 129.41, 128.49, 128.07, 127.89, 111.97, 111.64, 108.10, 66.81, 60.82, 56.31, and 55.67.

Compound **8**: Yield: 30.7% ESI-MS for C_23_H_26_Br_2_O_6_ (*m*/*z*): [M + Na]^+^ 581. ^1^H NMR (401 MHz, CDCl_3_) δ 7.72 (s, 1H), 7.47 (d, *J* = 2.1 Hz, 1H), 7.14 (dd, *J* = 8.4, 2.1 Hz, 1H), 6.95–6.92 (m, 1H), 6.35 (s, 2H), 4.22 (t, *J* = 6.2 Hz, 2H), 3.90 (s, *J* = 2.4 Hz, 3H), 3.81 (s, 3H), 3.58 (s, 6H), 3.38 (t, *J* = 6.5 Hz, 2H), 1.94–1.86 (m, 2H), and 1.86–1.78 (m, 2H). ^13^C NMR (101 MHz, CDCl_3_) δ 167.34, 155.44, 152.64, 140.74, 134.59, 130.08, 129.67, 129.62, 129.37, 112.00, 111.63, 108.11, 64.29, 60.80, 56.32, 55.67, 33.03, 29.38, and 27.17.

Compound **9**: Yield: 34.3% ESI-MS for C_25_H_30_Br_2_O_6_ (*m*/*z*): [M + Na]^+^ 609. ^1^H NMR (401 MHz, CDCl_3_) δ 7.71 (s, 1H), 7.47 (d, *J* = 2.1 Hz, 1H), 7.14 (dd, *J* = 8.4, 2.1 Hz, 1H), 6.61 (s, 1H), 6.34 (s, 2H), 4.19 (t, *J* = 6.5 Hz, 2H), 3.90 (s, 3H), 3.81 (s, 3H), 3.58 (s, 6H), and 1.88–1.14 (m, 8H). ^13^C NMR (101 MHz, CDCl_3_) δ 167.41, 155.38, 152.61, 140.50, 134.63, 131.07, 130.34, 130.10, 129.91, 129.72, 129.44, 126.50, 111.90, 111.73, 108.07, 105.37, 33.57, 32.49, 28.32, 27.58, and 25.05.

#### 4.1.5. General Procedure for the Synthesis of Compounds **10**–**22**

Compound **3** (1.0 eq) was dissolved in CH_2_Cl_2_ (20 mL) and cooled in an ice-water bath, then DCC (1.2 eq) was added. After 20 min, the solution of HOBt in 5 mL of THF was added (0.5 eq). After 10 min a respective amine was added. The reaction mixture was stirred for 24 h. Then, the precipitated DCU was separated under reduced pressure and the filtrate was evaporated to dryness in the presence of silica gel. The residue was purified by column flash chromatography using CombiFlash^®^Rf+ (chloroform/ethyl acetate, increasing concentration gradient) with an integrated Evaporative Light Scattering Detector (ELSD) and UV detector.

Compound **10**: Yield: 31.2% ESI-MS for C_20_H_22_BrNO_5_ (*m*/*z*): [M + Na]^+^ 458. ^1^H NMR (401 MHz, CDCl_3_) δ 7.71 (s, 1H), 7.48 (d, *J* = 2.1 Hz, 1H), 7.16 (dd, *J* = 8.4, 2.1 Hz, 1H), 6.97 (d, 1H), 6.24 (s, 2H), 3.89 (s, 3H), 3.76 (s, 3H), 3.54 (s, 6H), and 2.83 (d, *J* = 4.9 Hz, 3H). ^13^C NMR (101 MHz, CDCl_3_) δ 167.24, 155.95, 152.51, 137.29, 134.68, 131.54, 130.26, 129.81, 129.63, 112.69, 107.61, 60.69, 56.31, 55.51, and 26.92.

Compound **11**: Yield: 34.7% ESI-MS for C_23_H_28_BrNO_5_ (*m*/*z*): [M + Na]^+^ 500. ^1^H NMR (401 MHz, CDCl_3_) δ 7.71 (s, 1H), 7.49 (d, *J* = 2.1 Hz, 1H), 7.17 (dd, *J* = 8.4, 2.1 Hz, 1H), 7.01–6.97 (m, 1H), 6.25 (s, 2H), 3.92 (s, 3H), 3.78 (s, 3H), 3.56 (s, 6H), 3.29 (dd, *J* = 13.1, 7.1 Hz, 2H), 1.47–1.39 (m, 2H), 1.32–1.21 (m, 2H), and 0.88 (t, *J* = 7.3 Hz, 3H). ^13^C NMR (101 MHz, CDCl_3_) δ 166.55, 155.97, 152.56, 137.33, 134.74, 131.78, 130.23, 129.92, 129.73, 112.69, 112.58, 107.64, 60.73, 56.34, 55.56, 39.88, 31.52, 19.98, and 13.65.

Compound **12**: Yield: 35.8% ESI-MS for C_22_H_22_BrNO_5_ (*m*/*z*): [M+Na]^+^ 482. ^1^H NMR (401 MHz, CDCl_3_) δ 7.74 (s, 1H), 7.51 (d, *J* = 2.1 Hz, 1H), 7.19 (dd, *J* = 8.4, 2.1 Hz, 1H), 7.00 (d, 1H), 6.26 (s, 2H), 4.10 (dd, *J* = 5.4, 2.6 Hz, 2H), 3.92 (s, 3H), 3.78 (s, 3H), 3.56 (s, 6H), and 2.19 (t, *J* = 2.5 Hz, 1H). ^13^C NMR (101 MHz, CDCl_3_) δ 166.30, 156.12, 152.59, 138.35, 134.78, 130.85, 130.30, 129.60, 129.12, 112.78, 112.70, 107.78, 79.36, 71.51, 60.75, 56.36, 55.58, and 29.76.

Compound **13:** Yield: 39.6% ESI-MS for C_26_H_26_BrNO_5_ (*m*/*z*): [M] 512, [M + Na]^+^ 536, [M + K]^+^ 551. ^1^H NMR (401 MHz, CDCl_3_) δ 7.79 (s, 1H), 7.53 (d, *J* = 2.1 Hz, 1H), 7.32–7.28 (m, 2H), 7.27–7.22 (m, 3H), 7.20 (dd, *J* = 8.3, 2.0 Hz, 2H), 6.97 (d, 1H), 6.28 (s, 2H), 4.53 (d, *J* = 5.9 Hz, 2H), 3.90 (s, 3H), 3.80 (d, *J* = 2.1 Hz, 3H), and 3.57 (s, 6H). ^13^C NMR (101 MHz, CDCl_3_) δ 166.68, 156.04, 152.60, 138.15, 137.99, 134.78, 131.46, 130.25, 129.80, 129.44, 128.61, 127.37, 127.33, 112.76, 112.67, 107.71, 60.77, 56.33, 55.59, and 44.01.

Compound **14**: Yield: 40.9% ESI-MS for C_25_H_24_BrNO_5_ (*m*/*z*): [M + Na]^+^ 520. ^1^H NMR (401 MHz, CDCl_3_) δ 7.84 (s, 1H), 7.61 (d, *J* = 2.1 Hz, 1H), 7.48 (dt, *J* = 8.7, 1.7 Hz, 3H), 7.31–7.27 (m, 4H), 7.12–7.07 (m, 1H), 7.07–7.04 (d, 1H), 6.30 (s, 2H), 3.96 (s, 3H), 3.81 (s, 3H), and 3.59 (s, 6H).^13^C NMR (101 MHz, CDCl_3_) δ 164.61, 156.32, 152.66, 138.68, 137.69, 134.95, 131.87, 130.44, 129.68, 129.13, 128.88, 124.48, 119.91, 112.94, 112.90, 107.86, 60.78, and 56.40, 55.62.

Compound **15**: Yield: 25.4% ESI-MS for C_25_H_24_BrNO_5_ (*m*/*z*): [M] 557, [M + Na]^+^ 581, [M + K]^+^ 595. ^1^H NMR (401 MHz, CDCl_3_) δ 8.00 (dd, *J* = 8.1, 1.2 Hz, 1H), 7.70 (s, 1H), 7.66–7.58 (m, *J* = 9.0, 7.7, 1.5 Hz, 2H), 7.47 (d, *J* = 2.1 Hz, 1H), 7.46–7.41 (m, 1H), 7.15 (dd, *J* = 8.4, 2.1 Hz, 1H), 7.00 (d, 1H), 6.39 (t, *J* = 6.4 Hz, 1H), 6.25 (s, 2H), 4.71 (d, *J* = 6.4 Hz, 2H), 3.91 (s, 4H), 3.78 (s, 3H), and 3.55 (s, 7H). ^13^C NMR (101 MHz, CDCl_3_) δ 166.84, 156.09, 152.57, 138.04, 134.67, 133.87, 133.40, 132.01, 131.24, 130.04, 129.63, 129.12, 128.59, 125.01, 112.75, 112.70, 107.76, 60.73, 56.34, 55.57, and 41.88.

Compound **16**: Yield: 23.6% ESI-MS for C_26_H_25_BrClNO_5_ (*m*/*z*): [M + Na]^+^ 570. ^1^H NMR (401 MHz, CDCl_3_) δ 7.75 (s, 1H), 7.53 (d, *J* = 2.1 Hz, 1H), 7.37–7.31 (m, 3H), 7.24–7.18 (m, 4H), 7.00–6.98 (m, 1H), 6.27 (s, 2H), 4.57 (d, *J* = 6.1 Hz, 2H), 3.92 (s, 3H), 3.79 (s, 3H), and 3.57 (s, 6H). ^13^C NMR (101 MHz, CDCl_3_) δ 166.69, 156.12, 152.65, 138.04, 135.47, 134.84, 131.43, 130.31, 130.04, 129.82, 129.52, 129.44, 128.86, 127.03, 112.78, 112.73, 107.80, 60.82, 56.42, 55.65, and 42.31.

Compound **17**: Yield: 25.1% ESI-MS for C_26_H_25_BrClNO_5_ (*m*/*z*): [M+Na]^+^ 570. ^1^H NMR (401 MHz, CDCl_3_) δ 7.78 (s, 1H), 7.55–7.53 (m, 1H), 7.24–7.19 (m, 5H), 7.13–7.10 (m, 1H), 6.99 (d, *J* = 8.4 Hz, 1H), 6.28 (s, 2H), 4.49 (d, *J* = 6.1 Hz, 2H), 3.91 (s, 3H), 3.80 (d, *J* = 1.1 Hz, 3H), and 3.58 (s, 6H). ^13^C NMR (101 MHz, CDCl_3_) δ 166.83, 156.18, 152.68, 140.37, 138.34, 134.84, 131.26, 130.31, 129.92, 129.75, 129.41, 127.56, 127.53, 125.54, 112.88, 112.82, 107.86, 60.82, 56.42, 55.66, and 43.47.

Compound **18**: Yield: 26.7% ESI-MS for C_26_H_25_BrClNO_5_ (*m*/*z*): [M + Na]^+^ 570. ^1^H NMR (400 MHz, CDCl_3_) δ 7.70 (s, 1H), 7.46 (d, *J* = 2.1 Hz, 1H), 7.22–7.07 (m, 6H), 6.93–6.90 (m, 1H), 6.20 (s, 2H), 4.40 (d, *J* = 6.0 Hz, 2H), 3.84 (s, 3H), 3.73 (s, 3H), and 3.50 (s, 6H). ^13^C NMR (101 MHz, CDCl_3_) δ 166.74, 156.07, 152.59, 138.69, 138.21, 136.75, 134.75, 133.10, 131.20, 130.23, 129.69, 129.32, 128.92, 128.79, 128.75, 128.71, 112.77, 112.71, 107.71, 60.77, 56.36, 56.34, 55.57, and 43.32.

Compound **19**: Yield: 35.9% ESI-MS for C_25_H_32_BrNO_5_ (*m*/*z*): [M]^+^ 506, [M + Na]^+^ 528, [M+K]^+^ 544. ^1^H NMR (401 MHz, CDCl_3_) δ 7.57 (d, *J* = 2.1 Hz, 1H), 7.27 (dd, *J* = 8.2, 1.9 Hz, 1H), 6.83–6.80 (m, 1H), 6.50 (s, 1H), 6.36 (s, 2H), 3.86 (s, 3H), 3.80–3.79 (m, 3H), 3.61 (s, 6H), 3.27 (d, *J* = 41.7 Hz, 4H), 1.43–1.25 (m, 4H), and 0.93–0.76 (m, 6H).^13^C NMR (101 MHz, CDCl_3_) δ 170.95, 155.45, 152.76, 137.84, 135.97, 133.51, 130.44, 129.25, 129.18, 129.06, 111.67, 111.43, 106.55, 60.77, 56.18, 55.80, 50.24, 24.88, and 11.26.

Compound **20**: Yield: 37.4% ESI-MS for C_27_H_36_BrNO_5_ (*m*/*z*): [M]^+^ 534, [M + Na]^+^ 556. ^1^H NMR (600 MHz, CDCl_3_) δ 7.52 (d, *J* = 2.2 Hz, 1H), 7.22 (dd, *J* = 8.5, 2.2 Hz, 1H), 6.77–6.76 (m, 1H), 6.46 (s, 1H), 6.31 (s, 2H), 3.81 (s, 3H), 3.75 (d, *J* = 1.9 Hz, 3H), 3.57 (s, 6H), 3.34–3.16 (m, 4H), and 1.32–0.75 (m, 14H). ^13^C NMR (151 MHz, CDCl_3_) δ 170.89, 155.51, 152.83, 136.03, 133.60, 130.53, 129.25, 129.07, 111.71, 111.48, 106.55, 60.84, 56.25, 55.84, 48.43, 33.90, 19.96, and 13.81.

Compound **21**: Yield: 32.2% ESI-MS for C_23_H_26_BrNO_6_ (*m*/*z*): [M + Na]^+^ 514. ^1^HNMR (400 MHz, CDCl_3_) δ 7.51 (d, *J* = 2.2 Hz, 1H), 7.21 (dd, 1H), 6.79 (d, 1H), 6.52 (s, 1H), 6.32 (s, 2H), 3.82 (s, 3H), 3.75 (s, 3H), and 3.61–3.48 (m, *J* = 20.0 Hz, 14H). ^13^C NMR (101 MHz, CDCl_3_) δ 169.79, 155.55, 152.74, 137.96, 134.14, 133.40, 130.42, 129.94, 129.18, 128.85, 111.78, 111.57, 106.51, 66.61, 60.77, 56.19, 56.18, 55.75, and 55.75.

Compound **22**: Yield: 19.4% ESI-MS for C_33_H_32_BrNO_5_ (*m*/*z*): [M]^+^ 602, [M + Na]^+^ 624. ^1^H NMR (600 MHz, CDCl_3_) δ 7.53 (s, 1H), 7.28–7.18 (m, 11H), 6.72 (d, 1H), 6.52 (s, 1H), 6.18 (s, 2H), 4.57 (s, 2H), 4.40 (s, 2H), 3.80 (s, 3H), 3.73 (s, 3H), and 3.53 (s, 6H). ^13^C NMR (151 MHz, CDCl_3_) δ 171.70, 155.64, 152.79, 134.87, 133.64, 130.14, 129.99, 129.43, 128.82, 128.69, 127.51, 111.79, 111.67, 106.56, 60.83, 56.27, 55.83, and 29.64.

### 4.2. X-ray Measurements

The X-ray intensity data for the crystal **7** were collected using graphite monochromatic MoKα radiation on a four-circle κ geometry Xcalibur, Atlas diffractometer with a two-dimensional area CCD detector. The ω-scan technique with Δω = 1.0° for each image was used for data collection (Table 6). The data were collected using the CrysAlis CCD program [21]. Integration, the scaling of the reflections, correction for Lorenz and polarization effects, and absorption corrections were performed using the CrysAlis Red program [21]. The structures were solved by the direct methods using SHELXT [22] and refined using the SHELXL-SHELXL-2018/3 program [23]. The positions of the hydrogen atoms involved in the hydrogen bonds were located in difference Fourier maps and were refined with U_iso_ = 1.2U_eq_ of N joined H or U_iso_ = 1.5U_eq_ of O atom joined H. The hydrogen atoms linked to aromatic carbon atoms were introduced in their geometrical positions and treated as rigid. The final difference Fourier maps showed no peaks of chemical significance. The details of the data collection parameters, crystallographic data, and final agreement parameters are collected in Table 2. The visualizations of the structure were made with the Diamond 3.0 program [24]. The interactions between the molecules in the crystal were analyzed using the Hirshfeld surface analyses, and 2D fingerprint plots using the Crystal Explorer Ver. 3.1 program package [25]. For further details, see the crystallographic data for this compound deposited at the Cambridge Crystallographic Data Centre. Deposition number (https://www.ccdc.cam.ac.uk/services/structures (accessed on 10 April 2024)) CCDC No. 2338093 contains the supplementary crystallographic data for this paper.

### 4.3. In Vitro Biological Studies

#### 4.3.1. Cell Culture

The antiproliferative activity was tested on human primary (SW480) and metastatic (SW620) colon cancer, human prostate cancer (PC3), human liver cancer (HepG2), human breast cancer (MDA), human lung cancer (A549), and human immortalized keratinocyte (HaCaT) cell lines obtained from the American Type Culture Collection (ATCC, Rockville, MD, USA). The cells were cultured in EMEM (SW480 and SW620), RPMI 1640 (PC3) or DMEM High Glucose (HepG2, MDA, A549, and HaCaT) with stable glutamine supplemented with 10% heat-inactivated fetal bovine serum (FBS), penicillin (100 U/mL), and streptomycin (100 µg/mL) in 37 °C and 5% CO_2_/95% air humified incubator. The cells were grown until 80–90% confluence and then were harvested with 0.25% trypsin and used for the experiments. All the reagents for the cell culture were provided by VWR Chemicals.

#### 4.3.2. Cytotoxicity Assay

Cytotoxicity was tested with the sulforhodamine B (SRB) assay according to the National Cancer Institute protocol with slight modifications [26,27]. Briefly, cells were seeded on a 96-well plate (5 × 10^3^ cells per well). After 24 h incubation, zero-time control cells were fixed with TCA (final concentration 5%) for 1 h at 4 °C. The rest of the cells were treated with various concentrations (1–100 μM) of the tested compounds or reference compounds (colchicine, podophyllotoxin, and combretastatin A4 (CA4)) and incubated for 48 h at 37 °C and 5% CO_2_/95% air humified incubator. The untreated cells were used as the control. After incubation, the cells were fixed with TCA (final concentration 5%) for 1 h at 4 °C, gently washed 4 times with tap water, and left for drying. The dried fixed cells were incubated with SRB solution (0.05% SRB in 1% acetic acid) for 30 min rt, washed four times with 1% acetic acid, and left for drying. The dried cells were dissolved in 10 mM Tris-base solution (pH 10.5) and the absorbance was read at 510 nm.

The cell viability was presented as a percent of SRB reduction in the treated cells versus that in control cells. The mean absorbance for control cells was assumed as 100%. No-cell control (blank well) absorbance was assumed as 0%, unless the compound was cytostatic, then 0% referred to zero-time control. The IC_50_ values were calculated using the GraphPad Prism 10.1.2 (324) (USA) software. The most cytotoxic compounds (**8** and **20**) were selected for further in vitro experiments.

#### 4.3.3. Cell Cycle Analysis

The cell cycle was assessed for the compounds **8** and **20** and the combretastatin CA4 as a reference compound. The compounds were most cytotoxic to the MDA and A549 cancerous cell lines; thus, these cell lines were selected for further analyses. The HaCaT cell line was chosen to represent non-cancer cells. For the experiment, the cells were seeded on 6-well plates (5 × 10^4^ cells per well). After 24 h of incubation, the cells were treated with the compounds in selected concentrations (1.5 and 2 nM for CA4; 25, 30, and 40 mM for 8; and 15, 20, and 30 mM for 20) and incubated for the next 24 h. Then, the cells were detached with 0.25% trypsin, washed with 0.9% NaCl, and fixed with 70% ethanol at 4 °C for at least 16 h. For drying, the cells were washed twice with 0.9% NaCl, resuspended in 50 μg/mL propidium iodide (PI) and 100 μg/mL RNase in PBS and incubated for 30 min at 37 °C. The cell cycle distribution was analyzed by flow cytometer (FACS Verse, Becton, Dickinson and Company, Franklin Lakes, NJ, USA).

#### 4.3.4. Tubulin Polymerization

The assembly of purified bovine tubulin was monitored using a BK004P kit, purchased from Cytoskeleton Inc. (Denver, CO, USA). The assay was performed according to the manufacturer’s instructions using the standard assay conditions. Briefly, purified (>97%) porcine tubulin protein (3 mg/mL) in a buffer composed of 80 mM PIPES (pH 6.9), 0.5 mM EGTA, 2.0 mM MgCl_2_, 1 mM GTP, and 5% glycerol, was incubated at 37 °C in the presence of either vehicle (1% (*v*/*v*) DMSO—control wells), or CA-4 (5 μM) or colchicine (5 μM), or the compounds **8** and **20** (20 or 100 μM). Light was scattered proportionally to the concentration of the polymerized microtubules in the assay. Then, the tubulin assembly was monitored by turbidometry at 340 nm in a MultiscanGo spectrophotometer (ThermoFisher Scientific, Carlsbad, CA, USA) for 60 min. The time dependence of the tubulin polymerization was presented in the form of graphs prepared in the GraphPad Prism 8.4.3.686 (Boston, MA, USA) software.

### 4.4. Computational Studies

#### 4.4.1. Homology Modeling of Human Tubulin Isotypes

The atomic coordinates of human tubulin isotypes were obtained by homology modeling based on the same template. To choose the best template, three crystallographic structures of bovine tubulin were selected from the Protein Data Bank (PDB): 1SA0, 4O2B, 5LYJ [28,29,30,31]. These structures were then compared according to their resolution, the number of missing residues (MRESs), and publication date.

As 1SA0 and 4O2B refer to a tubulin–colchicine complex, 5LYJ is a tubulin–CA-4 complex, so the 5LYJ crystal structure was chosen. This crystal structure, in addition to containing CA-4 at the colchicine site, is resolved at high resolution, has a low number of missing residues, and is relatively recent.

This derived structure has an intrinsically mobile C-terminal domain, so no crystallographic information on these regions is available [32]. In addition, these regions are not involved in the colchicine binding site, so including them in the modeling process would not alter the overall binding quality of the drugs with tubulin. Consequently, the carboxy terminus of the sequence was not included in the calculations.

It is important to take into account that unprepared files, such as PDB files, may contain missing atoms, alternate geometry, or other crystallographic artifacts; therefore, the 5LYJ structure was prepared using the QuickPrep command on MOE (Molecular Operating Environment) software package, with default settings. Running QuickPrep ensures a reasonably good quality of starting data by deleting distant solvents, adding hydrogens, installing tethers, calculating charges, and performing the initial refinement of the system.

The crystallographic structure presents two αβ heterodimers. Chain A and B, containing GTP, Mg^2+^, and solvent atoms in α-tubulin and GDP, Mg^2+^, and solvent atoms in β-tubulin, and CA-4 were selected to perform the homology modeling. Other chains and ligands were removed from the crystal structure by simply deleting them from the system manager panel in MOE.

#### 4.4.2. Human Sequence Selection

The human sequences of the α and β-tubulin isotypes were retrieved from the UniProt protein database [33]. They are αIa (Q71U36), βI (P07437), βIIa (Q13885), βIIb (Q9BVA1), βIII (Q13509), βIVa (P04350), and βIVb (P68371) [32]. To choose the correct sequences from the UniProt protein database the following gene nomenclature was used: TUBB (βI), TUBB2A (βIIa), TUBB2B (βIIb), TUBB3 (βIII), TUBB4 (βIVa), and TUBB2C (βIVb) [18].

As mentioned above, the crystal structure 5LYJ.pdb was taken as a reference template structure to build the 3D homology models of human αIa, βI, βIIa, βIIb, βIII, βIVa, and βIVb-tubulin isotype. Multiple sequence analyses of the human tubulin isotypes against the template sequence were performed to identify the residue composition variations. The percentage of identity between the human sequences and the bovine template ones was evaluated using the Sequence Editor of MOE. To evaluate which amino acid was different between the human β-tubulin isotypes, the sequences were uploaded on the ClustalΩ software package to perform the sequence alignment [34].

## 5. Conclusions

To sum up, a novel series of six esters and thirteen amides of CA4 was synthesized by the modification of the ethene bridge. The CA4 derivatives were obtained with moderate yields by two well-known esterification methods and one amidation method. According to the X-ray crystal analysis of the benzyl ester **7**, the whole molecule exhibits a non-planar conformation, but has planar fragments. Furthermore, in vitro biological activity of the obtained analogs was tested using six human cancer cell lines and one non-cancer keratinocyte cell line. The IC_50_ values were measured and compared with those describing the activity of the three reference compounds. It has been shown that the new derivatives exhibit lower antiproliferative activity than the unmodified CA4, colchicine, and podophyllotoxin. Among them, the compounds **8** and **20** appeared to be the most cytotoxic. Our computational analysis provided a reasonably good correlation between the experimental in vitro results and the binding affinities calculated by the docking technique based on the use of the weighted averages that account for the differential expression of the different tubulin isotypes in different cell lines. On the other hand, none of the tested ADMET (pharmacokinetic) descriptors correlated well with the experimental data. The only parameter that improves the linear regression model between binding energy weighted by the expression of the β tubulin isotypes and LogIC_50_ is the topological polar surface area (TPSA) computed for each of the compounds. Including TPSA as an additional descriptor (together with the binding affinity), the coefficient of determination (R2) has been found to reach values greater than 0.7 for some of the cell lines investigated here. The linear regression models based on assuming a uniform distribution of the isotypes led to a deterioration of the modeling accuracy resulting in lower R2 values. The most promising of the derivatives, the compounds **8** and **20**, produced the best results in vitro. The in silico analysis of the RMSD values for binding sites and the visualization of the trajectories for these compounds has led to the conclusion that they bind to beta tubulin at the same location as colchicine, i.e., at the interface between α and β tubulin, and that they are stably bound over time. Moreover, molecular dynamics simulations show that the compound **20** exhibits some selectivity for βIII; which is important in view of the overexpression of βIII tubulin in many cancer cell types.

The CA-4 molecule is composed of the three most important structural units. SAR studies have demonstrated that the following are crucial for tubulin polymerization inhibitory effects:

(a) 3,4,5-trimethoxyphenyl-subsituted ring A.
(b) 3-hydroxy-4-methoxyphenyl-substituted ring B.
(c) Cis double bond separating the two phenyl rings.

We think that the presence of the OH group instead of the Br atom could improve the activity. In this work we described for the first time a new method of synthesis, using microwave radiation, which proved to be an excellent procedure ensuring the formation of cis-stilbene isomer. Unfortunately, this method did not work in the case of the compounds with a free OH group (phenol). During the reaction, the OH group underwent esterification, and the product was often an oligomer. Knowing that a bromine atom is not the ideal bioisostere of the OH group, we decided to obtain a series of compounds with Br atoms to explore new methods of synthesis and the biological properties of the obtained compounds. We suppose that the skillful blocking and unblocking of the OH group may help us in the future in the synthesis of the compounds having the OH group instead of the Br atom in the final structure.

Theoretically, our compounds (amide and esters) can, especially in living organisms (in vivo), be hydrolyzed to an acidic form (compound **3**). This compound can interact with other target proteins (enzymes or receptors), but in vitro studies also show that the compound **3** does not have any anticancer potential. We did not observe hydrolysis in saline, but in compounds stored in sunlight for a long time, the isomerization of the double bond occurred.

## Figures and Tables

**Table 1:** Conformational details of the benzyl ester of **7**.

Torsion Angles			
C19—C20—O4—C24	−4.6 (7)	C23—C22—O6—C26	2.9 (6)
C22—C21—O5—C25	−101.6 (5)	C17—C8—C9—O2	5.3 (5)
C5—C8—C17—C18	−1.0 (7)	C8—C17—C18—C19	8.5 (6)
O2—C10—C11—C12	101.4 (5)	C6—C5—C8—C17	83.3 (5)
rings: C18 to C23 = Plane P1; C1 to C6 = Plane P2; C11 to C16 = Plane P3			
dihedral angles between the planes of the phenyl rings:			
P1/P2 = 77.3 (4)	P1/P3 = 69.3 (4)	P2/P3 = 14.8 (4)	

**Table 3:** Binding energy values for the combretastatin derivatives calculated using the MOE software for each tubulin isotype separately. The various tubulin isotypes are listed along the horizontal line while the compounds are listed vertically.

	αβI	αβIIa	αβIIb	αβIII	αβIVa	αβIVb	αβV	αβVI	αβVIII
**4**	−8.69	−8.35	−8.4	−8.67	−8.63	−8.52	−8.21	−8.81	−8.9
**5**	−9.32	−8.96	−8.95	−9.16	−9.21	−9.1	−8.8	−9.5	−9.46
**8**	−7.83	−7.66	−7.57	−7.85	−7.79	−7.72	−7.12	−7.98	−7.9
**10**	−8.56	−8.51	−8.53	−8.34	−8.78	−8.63	−8.34	−8.44	−8.58
**11**	−9	−8.82	−8.84	−8.86	−9.16	−9.14	−8.82	−8.89	−8.92
**13**	−8.67	−8.48	−8.16	−8.99	−8.85	−8.49	−8.47	−8.92	−8.98
**16**	−9.56	−8.91	−9.23	−8.95	−9.47	−9.32	−9.12	−9.49	−8.98
**18**	−8.52	−9.63	−9.36	−9.36	−9.57	−9.73	−8.92	−9.28	−9.2
**20**	−7.94	−7.59	−7.57	−7.76	−7.98	−8.13	−8.06	−7.82	−7.59

**Table 4:** Binding energy values calculated using weighted averages for individual tubulin isotypes corresponding to the expression level in the seven cell lines tested experimentally.

	SW480	SW620	PC3	HepG2	MDA	A549	HaCaT
**4**	−8.10	−8.16	−8.09	−8.14	−8.04	−8.08	−8.08
**5**	−8.43	−8.49	−8.43	−8.48	−8.38	−8.41	−8.41
**8**	−7.68	−7.75	−7.67	−7.75	−7.62	−7.65	−7.69
**10**	−8.48	−8.52	−8.48	−8.51	−8.44	−8.47	−8.44
**11**	−8.56	−8.60	−8.55	−8.58	−8.52	−8.55	−8.52
**13**	−8.47	−8.53	−8.47	−8.51	−8.42	−8.46	−8.42
**16**	−8.64	−8.67	−8.63	−8.62	−8.61	−8.63	−8.58
**18**	−8.56	−8.60	−8,56	−8.54	−8.53	−8.55	−8.50
**20**	−8.46	−8.50	−8.46	−8.47	−8.43	−8.45	−8.40

**Table 5:** Linear regression models: R^2^ value. BE*_w_*: weighted binding energy, MLogP: logarithm of the partition coefficient, TPSA: topological polar surface area, HBD: hydrogen bond donor, POL: polarizability.

R^2^	SW480	SW620	PC3	HepG2	MDA	A549	HaCaT
BE*_w_*	0.59	0.62	0.63	0.62	0.64	0.63	0.51
MLogP	3.1 × 10^−5^	1.4 × 10^−5^	1.8 × 10^−3^	3.1 × 10^−4^	4.7 × 10^−3^	5.2 × 10^−5^	2.5 × 10^−4^
TPSA	0.25	0.25	0.24	0.25	0.30	0.30	0.28
HBD	0.32	0.36	0.39	0.41	0.42	0.43	0.38
POL	7.5 × 10^−5^	4.4 × 10^−6^	1.4 × 10^−3^	4 × 10^−4^	5 × 10^−3^	1.6 × 10^−4^	7.6 × 10^−4^

**Table 6:** Crystallographic data and final refinement parameters for **7**.

Formula	C_26_H_25_BrO_6_
Molecular weight	513.37
Temperature (K)	295 (1)
Crystal system	monoclinic
Space group	*P2*_1_
*a* (Å)	7.8506(3)
*b* (Å)	14.7568(5)
*c* (Å)	10.2790(4)
β (°)	95.966(3)
*V* (Å^3^)	1184.37(8)
Z	2
*F*(000)	528
*D_cal_* (g cm^−1^)	1.440
θ range (°)	2.609 ÷ 27.500
μ (mm^−1^)	1.775
Crystal size (mm)	0.287 × 0.242 × 0.203
T_min_/T_max_	0.8123/1.000
Total/unique/obs refls	17727/5295/4113
*R_int_*	0.030
*R* [*F*^2^ *> 2σ*(*F*^2^)] *^a^*	0.0377
*wR* [*F*^2^ all refls] *^a^*	0.0762
Flack parameter	0.006(4)
*S*	1.002
Δρ_max_, Δρ_min_ (eÅ^−3^)	+0.224, −0.278

*^a^ R* = Σ ||*F*_o_| − |*F*_c_||/Σ*F*_o_, *wR* = {Σ [*w*(*F*_o_^2^ − *F*_c_^2^)^2^]/Σ*wF*_o_^4^}^½^; *w*^–1^ = σ^2^(*F*_o_^2^) + (*aP*)^2^ + (*b*P) where *P* = (*F*_o_^2^ + 2*F*_c_^2^)/3. The *a* and *b* parameters are 0.0355 and 0.0818, respectively.

## Data Availability

Data are contained within the article.
